# New Insights Into Microbiota Modulation-Based Nutritional Interventions for Neurodevelopmental Outcomes in Preterm Infants

**DOI:** 10.3389/fmicb.2021.676622

**Published:** 2021-06-11

**Authors:** Sylvie Buffet-Bataillon, Amandine Bellanger, Gaelle Boudry, Jean-Pierre Gangneux, Mathilde Yverneau, Alain Beuchée, Sophie Blat, Isabelle Le Huërou-Luron

**Affiliations:** ^1^Institut NuMeCan, INRAE, INSERM, Univ Rennes, Saint-Gilles, France; ^2^Department of Clinical Microbiology, CHU Rennes, Rennes, France; ^3^Department of Pediatrics-Neonatology, CHU Rennes, Rennes, France; ^4^CHU Rennes, INSERM, Irset, UMR_S 1085, Univ Rennes, Rennes, France; ^5^Department of Pediatrics-Neonatology, Univ Rennes, CHU Rennes, LTSI-UMR 1099, Rennes, France

**Keywords:** microbiota, preterm, breast-feeding, infant formula, maternal nutrition in pregnancy

## Abstract

Gut microbiota and the central nervous system have parallel developmental windows during pre and post-natal life. Increasing evidences suggest that intestinal dysbiosis in preterm infants predisposes the neonate to adverse neurological outcomes later in life. Understanding the link between gut microbiota colonization and brain development to tailor therapies aimed at optimizing initial colonization and microbiota development are promising strategies to warrant adequate brain development and enhance neurological outcomes in preterm infants. Breast-feeding has been associated with both adequate cognitive development and healthy microbiota in preterms. Infant formula are industrially produced substitutes for infant nutrition that do not completely recapitulate breast-feeding benefices and could be largely improved by the understanding of the role of breast milk components upon gut microbiota. In this review, we will first discuss the nutritional and bioactive component information on breast milk composition and its contribution to the assembly of the neonatal gut microbiota in preterms. We will then discuss the emerging pathways connecting the gut microbiota and brain development. Finally, we will discuss the promising microbiota modulation-based nutritional interventions (including probiotic and prebiotic supplementation of infant formula and maternal nutrition) for improving neurodevelopmental outcomes.

## Introduction

The impact of the microbiota on mammalian development has been well documented with scientific evidence highlighting an association between gut microbiota and brain functions through the humoral and neural pathways of the gut-brain axis (Burokas et al., 2015; Cryan and Dinan, 2015). The intestinal microbiota is critical in the functional development of microglia, a key element in the prevention of neurodevelopmental and neurodegenerative diseases (Hazlett et al., 2005; Harry, 2013; Erny et al., 2015). As an example, modified levels of Firmicutes and Bacteroidetes were reported in the gut microbiota of children with autism (Finegold et al., 2010; Tomova et al., 2015). In addition, schizophrenia and attention-deficit hyperactivity disorders are associated with intestinal dysbiosis (Dinan et al., 2014; Cenit et al., 2017). The intestinal microbiota could affect brain physiology by modification of epigenetic modulation-based gene expression of genes associated with neuronal plasticity, learning, memory, and neurogenesis, as well as with behavioral disorders (Stilling et al., 2014; Majnik and Lane, 2015). Corroborating clinical research, animal studies have shown that germ-free mice exhibit disturbed social behavior and that brain morphological organization and development rely on gut microbiota composition (Diaz Heijtz et al., 2011; Desbonnet et al., 2014; Lu et al., 2018).

Survival of preterm babies has increased worldwide, with a concomitant reduction in severe neonatal morbidity. However, a recent evaluation of developmental and behavioral outcomes in a large French cohort of preterm infants, EPIPAGE, clearly highlighted that 40–50% (at 2 years of corrected age) and 19–28% (at 5 years of corrected age) of neonates born at less than 32 weeks of gestational age displayed neurodevelopmental delays (Pierrat et al., 2017, 2020). This study corroborates other works reporting language difficulties up to 13 ears of age in children born preterm, with no evidence of developmental “catch-up” (Diaz Heijtz et al., 2011; van Noort-van der Spek et al., 2012; Nguyen et al., 2018) and disabilities such as school difficulties and behavioral problems that emerged into adolescence (Saigal and Doyle, 2008). Children with such delays may represent a group at risk for future academic difficulties and with poorer social-emotional competence although most of them recover well during their transition into adulthood (Saigal and Doyle, 2008). Moreover, an immature microbiota was associated with 2-year non-optimal neurodevelopmental outcomes in preterm infants (Rozé et al., 2020). The goal of early perinatal intervention is therefore to reduce or prevent abnormal brain development. Recent studies revealed how maternal nutrition during pregnancy and nursing and infant formula feeding influenced both offspring microbiota and brain neurogenesis, and later cognitive and behavior (eating, social, locomotor, and exploratory) abilities (Ehrenkranz et al., 2006; Van Lieshout, 2013). Targeting the critical window of both gut microbiota and brain early development with personalized nutrition to apply potential neuroprotective strategies has potential therapeutic significance for preterm infants (Diaz Heijtz et al., 2011; Borre et al., 2014a, b).

In this review, we will describe the composition of preterm breast milk and infant formula for preterm infants, the development of the gut microbiota, and the neurodevelopment deficits associated with altered gut microbiota, and discuss potential new therapeutic strategies to restore microbiota and optimize neurodevelopment in preterm infants.

## Composition of Preterm Infant Feeding: Breast Milk and Infant Formula

### Human Milk Macronutrient Composition: Preterm vs. Term

Adequate nutritional supply during the first weeks of life is critical for neurodevelopment and growth of preterm infants (Coviello et al., 2018). Breast milk is the optimal diet for term infants as well as for premature infants as early as their digestive system is mature for macronutrient digestion. Benefits of human milk (HM) to preterm neonates include improvements in host defense, digestion and absorption of nutrients, gut function and neurodevelopment, short-term protection against necrotizing enterocolitis (NEC) and better long term health outcomes (Bauer and Gerss, 2011). However, suboptimal weight gain and nutritional deficits may be observed in premature babies born at a gestational age below 28 weeks, due to their requirements for large amounts of protein and energy to achieve appropriate growth (European Society of Paediatric Gastroenterology and Nutrition Committee on Nutrition of the Preterm Infant, 1987; Agostoni et al., 2010; Joosten et al., 2018). HM fortification in energy, proteins and minerals is therefore recommended in routine nutritional neonatal care of preterm infants.

The average macronutrient composition of mature (>4 weeks of lactation), term milk ranges approximately from 0.9 to 1.2 g protein/100 mL, 3.4 to 4.1 g fat/100 mL, and 6.2 to 7.4 g lactose/100 mL (Ballard and Morrow, 2013; Le Huërou-Luron et al., 2018). That of preterm milk after the 5th week of lactation ranges from 1.0 to 2.0 g protein/100 mL, 3.7 to 4.5 g fat/100 mL, and 7.5 g lactose/100 mL (Bauer and Gerss, 2011; Gidrewicz and Fenton, 2014). It is noteworthy that recent metabolomic analyses of milk revealed that lactose and HM oligosaccharide (HMO) levels were significantly greater in preterm than in term milk collected 1–3 weeks postpartum (Sundekilde et al., 2016; Perrone et al., 2019). The metabolizable energy content of term and preterm milk ranges from 61 to 92 and 48 to 85 kcal/100 mL, respectively, one-half provided by fat (Gidrewicz and Fenton, 2014). The macronutrient content of breast milk changes throughout the course of lactation. Both preterm and term colostrum has higher content of protein, but lower contents of energy, fat and lactose than mature milk (Bauer and Gerss, 2011; Gidrewicz and Fenton, 2014; Bulut et al., 2019). HM contains hundreds of bioactive molecules that considerably contribute to overall health benefits for neonates (Carr et al., 2021). Its composition in bioactive molecules changes between mothers in relation with the degree of prematurity, genetic and dietary factors.

#### Protein Content in Preterm and Term Breast Milk (Figure 1)

**FIGURE 1:**
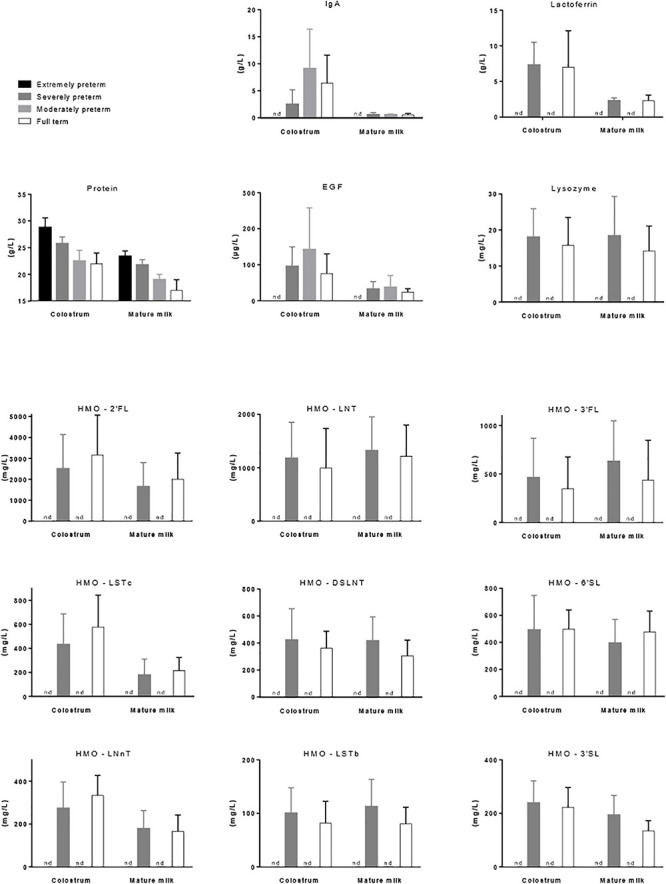
Mean concentration (±*SD*) of protein (Bauer and Gerss, 2011), IgA (Castellote et al., 2011), lactoferrin (Mastromarino et al., 2014), EGF (Castellote et al., 2011), lysozyme (Hsu et al., 2014), and nine prevalent forms of HMOs (Austin et al., 2019) in the colostrum (<7 days of lactation) and mature milk (1 month of lactation) of mothers that gave birth to extremely preterm (below 29 weeks of gestation), severely preterm (28–32 weeks, excepted for lactoferrin 26–36 weeks), moderately preterm (30–37 weeks) or full term (36–42 weeks) babies. nd, not determined.

It is noticeable that milk produced by mothers who deliver prematurely has higher protein concentration during the first weeks of lactation than the milk of mother who delivered at term. A meta-analysis including forty-one studies on the nutrient content of preterm (<37 weeks of gestation) and term (37–42 weeks of gestation) 24-h collected HM reported up to 35% (0.7 g/100 mL) higher true protein content in preterm colostrum than term colostrum (Gidrewicz and Fenton, 2014). Also of importance, the inter-individual variability of the protein content in preterm milk in the first postnatal days is higher than in term milk (Gidrewicz and Fenton, 2014; Fischer Fumeaux et al., 2019). Thereafter, most of the differences in true protein between preterm and term milks were within 0.2 g/100 mL with average protein concentration of 1.4 g/100 mL and 1.2 g/100 mL at 3–4 weeks of lactation, respectively. No more differences occurred beyond postnatal weeks 10–12. However, the previous longitudinal analysis of Bauer and Gerss (2011) reported substantially higher protein content values (Figure 1). More precisely, protein concentration differed by 0.19 g/100 mL (average value on the first 8 weeks of gestation) in extremely preterm milk (<28 weeks of gestation) vs. severely preterm milk (28–31 weeks of gestation), by 0.45 g/100 mL in extremely preterm milk vs. moderately preterm milk (32–33 weeks of gestation), and by 0.73 g/100 mL in extremely preterm milk vs. term milk (Bauer and Gerss, 2011).

Besides their critical importance in terms of nutrition and source of amino acids, proteins have potential effects on the developing microbiota of neonates. Although milk protein digestibility is very high, undigested dietary protein or fractions of these proteins, associated with endogenous proteins may be fermented by preterm gut microbiota (Boudry et al., 2016; Beverly et al., 2019). Indeed, for low birth weight piglets characterized with intestinal immaturity, the quantity of protein intake, specifically when fed formula with high protein level, induced sustained modifications of gut microbiota composition in the first and 5 months of age (Chatelais et al., 2011; Boudry et al., 2013).

Colostrum is rich in immunologic and developmental factors such as immunoglobulins (Igs), predominantly IgA, epidermal growth factor (EGF), TGFβ1, TGFβ2 and cytokines (IL-6, IL-8, IL-10, IL-13, and TNFα), supporting the maturation of the neonatal intestine and its immune system. The decrease in immunologic component concentration observed during lactation was described to be dependent on gestational length (Castellote et al., 2011). The colostrum of preterm women (delivery between weeks 30 and 37 of gestation) was equal to or richer than that of term women (delivery between weeks 38 and 42 of gestation) for the above-mentioned immunologic factors. Conversely, the colostrum of very preterm women (delivery before week 30 of gestation) had lower contents in most of the immunologic factors than that of term women (Figure 1). HM is the main source of IgA for neonates as they produce very low amounts of IgA during the first 2 weeks of life. Therefore, after birth, HM composition doesn’t allow effective immune defense for women with very preterm babies as opposed to women with preterm babies. At the end of the first month of lactation, immunologic factor concentrations were similar in the mature milk of women, whatever their gestational length, suggesting that only the colostral immunologic supply is dependent of childbirth date (Castellote et al., 2011).

Among proteins present in breast milk, some have anti-microbial properties including IgA, lactoferrin and lysozyme. IgA plays an essential role in defense against pathogenic bacteria but also in controlling gut microbiota composition. Indeed, selective IgA-deficiency in adult humans induced an altered gut microbiota composition (decreased bacterial diversity and changes in abundances of specific bacterial groups such as increased relative abundances of Proteobacteria, notably of the inflammatory facultative anaerobes *Enterobacteriaceae*, and of some taxa of the *Ruminococcaceae* family) as compared to adult healthy controls (Catanzaro et al., 2019). In healthy condition, a great proportion (24–74%) of live bacterial cells is bound by IgA, reflecting a stable IgA response to commensal bacteria (Mantis et al., 2011). With the human commensal *Bacteroides fragilis*, it was demonstrated that IgA-bacteria interaction facilitated bacterial adherence to the gut mucosal surface and stable colonization of the gut through exclusion of exogenous competitors (Donaldson et al., 2018). The abundance of IgA-bound bacteria was greater in fecal samples from breast-milk-fed infants compared to formula-fed infants (Gopalakrishna et al., 2019). Moreover, the importance of the maternal IgA-bacteria interaction in the protection against NEC in preterms was recently confirmed (Gopalakrishna et al., 2019). Indeed, a relative decrease in IgA-bound bacteria and an increasing dominance held by *Enterobacteriaceae* in the IgA-unbound fraction of the microbiota was associated with the development of NEC in preterm neonates. In addition, Igs which are *N*-glycosylated proteins may be a carbon source for gut bacteria such as *Bacteroides* that express carbohydrate-active enzymes involved in the degradation of glycans (Briliute et al., 2019).

Lactoferrin is another HM component that favors gut infant colonization with beneficial bacteria (Mastromarino et al., 2014). The amount of lactoferrin in feces in 1-month old infants was positively associated with the amount of lactoferrin in mature HM, confirming that HM represents the main source of lactoferrin in infant gut. A bifidogenic effect of lactoferrin was reported *in vitro* and *in vivo* in both neonatal pigs and human infant microbiota-associated mice fed bovine lactoferrin-fortified milk (Hentges et al., 1992; Hu et al., 2012) as well as in human infants after 3 months feeding with a lactoferrin enriched formula (Roberts et al., 1992). Lactoferrin concentration decreased during the first month of lactation in both full-term (36–41 weeks of gestation) and pre-term (26–36 weeks of gestation) milks with no significant differences between full-term and pre-term milks (Mastromarino et al., 2014) (Figure 1). However, positive correlations between lactoferrin concentration and fecal *Bifidobacterium* and *Lactobacillus* were demonstrated at birth in preterm, but not term, neonates (Mastromarino et al., 2014). Therefore, lactoferrin may represent an important factor driving the composition of the neonatal gut microbiota particularly in preterm infants although its benefit in reducing mortality or significant neonatal morbidities in very low birth weight infants was not clearly demonstrated using bovine lactoferrin supplementation (Asztalos et al., 2020).

Lysozyme is present in breast milk at relatively high concentrations and degrades the outer cell walls of Gram-negative bacteria. Lysozyme content decreased progressively during the first 2 weeks of lactation, without any major differences in relation to the gestational age at delivery (Montagne et al., 1999; Hsu et al., 2014).

#### Lipid Content in Preterm and Term Breast Milk

Dietary lipids are crucial for neonates to meet their high energy requirements but also numerous physiological functions critical to their growth and health. The anatomical and functional development of the brain depends particularly on the supply of long-chain polyunsaturated fatty acids (LC-PUFAs). Human milk fat provided in the form of globules consists mainly of 97% triglycerides (TAG), small amounts of mono- and diacylglycerides (MAG and DAG), around 1% of phospholipids and 0.5% of sterols, mostly cholesterol (Bourlieu et al., 2015). HM lipid specificity consists of over 400 fatty acids (FAs) with variable chain lengths and unsaturations, an esterification of palmitic acid primarily at the sn-2 position (∼70%), unsaturated FAs mainly at the sn-1,3 positions and the high proportion of LC-PUFAs [*n*−6 (such as arachidonic acid, AA) and *n*−3 (such as eicosapentaenoic, EPA and docosahexaenoic acids, DHA), which are derived from the essential PUFAs linoleic acid (LA) and alpha-linolenic acid (ALA), respectively] (Le Huërou-Luron et al., 2018). The content in LA and ALA ranges from 10 to 24% of FAs and from 0.6 to 1.9% of FAs, respectively (Delplanque et al., 2015). Total milk fat and the essential PUFAs contents increase with milk maturation whereas the proportion of *n*−6 and *n*−3 LC-PUFAs decreases markedly by about 38% for AA and about 50% for DHA during the course of the first month of lactation (Koletzko et al., 2001). During the last trimester of gestation the supply of LC-PUFAs from maternal plasma to the fetus is actively provided by transfer across the placenta. Thus, in premature birth, the maternal FAs supply stops early and the preterm infant receives less amount of LC-PUFAs prior to birth than the full-term infant (Mazzocchi et al., 2018). Some studies reported slightly higher proportions of DHA and AA in preterm than term milk, but also of medium- and intermediate-chain length FAs, which could support the higher LC-PUFAs requirement of preterms at birth (Lapillonne et al., 2013), but others reported lower levels of EPA and DHA in preterm milk (Berenhauser et al., 2012), while others did not find any difference between preterm and term milk (Granot et al., 2016). Short- and medium-chain FAs (butyrate, caprylate and caprate) were found in lower concentrations in preterm milk compared with term milk (Sundekilde et al., 2016). HM of women who gave birth prematurely displayed a deficiency in vitamins A and E (Sámano et al., 2017).

Very little is known about the influence of milk FAs on the gut microbiota. *In vitro*, medium-chain FAs and digestion products of sphingolipids demonstrated bactericidal activities against pathogens, suggesting a potential protection against food-borne gastroenteritis (Sprong et al., 2001). Moreover, medium-chain FAs were shown to modify gut microbiota *in vitro* (Nejrup et al., 2015), in piglets (Zentek et al., 2012) and germ-free mice (Nejrup et al., 2017). In infant formula, increasing the proportion of palmitate esterified in the sn-2 position resulted in higher *Lactobacillus* and *Bifidobacterium* counts in fecal stools of term formula-fed infants to a level similar to that of breast-fed infants (Yaron et al., 2013). A significant association between sn-2 FAs in milk and infant gut microbiota was confirmed in a Chinese human cohort, particularly between C10:0 to C18:0 FAs, LC-PUFAs (AA and DHA) with *Bacteroides, Enterobacteriaceae, Veillonella, Streptococcus*, and *Clostridium* (Jiang et al., 2018). To our knowledge, such correlations in preterm milk have not been studied.

Beyond FA composition, the lipid matrix structure is also of great importance. HM fat is organized in its native form in dispersed globules enveloped by a tri-layer biological membrane called milk fat globule membrane (MFGM) which has been reported to provide a beneficial impact on human brain development, gut immunity and barrier functions in neonates when supplemented in infant formula (Timby et al., 2017b; Lemaire et al., 2018). MFGM composition is influenced by maternal factors and by the changing needs of the infant over the period of lactation (Lee et al., 2018), leading to the speculation that its composition may also change in preterm milk, although no data are currently available. Lipids, proteins and surface carbohydrate moieties in the MFGM might have an important role in profiling gut microbiota as thoroughly reviewed (Lee et al., 2018), although moderate effects have recently been observed on the oral microbiota of 4 month-old infants fed formula supplemented with a bovine MFGM concentrate compared to a standard formula (Timby et al., 2017a). Moreover, MFGM may influence protein digestion and consequently the protein digestion products that enter the colon, and by this way indirectly influence microbiota composition in the infant gut (Le Huerou-Luron et al., 2018). Data on the impact of prematurity on MGFM composition are scarce. Levels of total phospholipids and gangliosides in HM appeared to be highest in colostrum. Higher sphingomyelin and lower or similar phospholipid compositions have been reported in preterm milk compared to term milk (Bitman et al., 1984; Shoji et al., 2006), but Maas et al. (2017) recently reported that HM concentration of choline-containing compounds, including phosphatidylcholine and sphingomyelin, were lower after preterm compared to term delivery. Intriguingly, phosphatidylcholine precursors, choline and phosphocholine, were found in higher levels in preterm milk than in full term milk (Sundekilde et al., 2016).

#### Lactose and Human Milk Oligosaccharides (HMO) Content in Preterm and Term Breast Milk

Human milk oligosaccharides include more than 200 different compounds and form the third-largest solid component in HM after lipids and lactose (5.5–7.0 g/100 mL). Their concentration range from 2.5 g/100 mL in colostrum and between 1.0 and 1.5 g/00 mL in mature term milk (Kunz and Rudloff, 1993; Kunz et al., 1999). HMOs are complex glycans, non-hydrolyzed by gut digestive enzymes but a substrate for infant gut microbiota. HMOs prevent attachment of pathogens to the mucosa, as soluble decoy receptors for pathogens, and may reduce the presence of glycans expressed at the epithelial surface of the intestine (Kunz et al., 2000). HMOs also play roles in prevention of gut dysfunction. Specifically, lower breast milk content of disialyllacto-*N*-tetraose (DSLNT) which is linked with NEC prevention in neonatal rats, was associated with high NEC prevalence for human, as shown in a study of 200 American mothers with very low birth weight infants (birth weight under 1,500 g) (Autran et al., 2018). It is noticeable that the pattern of oligosaccharides in HM is highly variable between mothers. The presence or absence of functional fucosyltransferase-2 (FUT2) and fucosyltransferase-3 (FUT3) enzymes depending on maternal genetic factors is known to significantly influence the HMO profile. The mother’s Secretor and Lewis blood groups define four phenotypes: Lewis positive Secretors (FUT2 active, FUT3 active; Le+Se+), Lewis negative Secretors (FUT2 active, FUT3 inactive; Le−Se+), Lewis positive Non-secretors (FUT2 inactive, FUT3 active; Le+Se−) and Lewis negative Non-secretors (FUT2 inactive, FUT3 inactive; Le−Se−), characterized by different levels of HMOs. Infants of secretor mothers (Le+Se+ or Le−Se+) may be protected by fucosylated HMOs that decrease the levels of pathogens associated with NEC and sepsis (Bering, 2018). In addition, higher levels of Proteobacteria and lower levels of Firmicutes were observed in preterm infants of non-secretor mothers (Underwood et al., 2015). Other factors including lactation stage, parity, mode of delivery, pre-pregnancy maternal body mass index may also play a significant role in HMO composition (Azad et al., 2018; Samuel et al., 2019).

Discrepancies exist in the literature related to the prematurity effect on HMO content, i.e., no change in HMO composition (Kunz and Rudloff, 1993; Nakhla et al., 1999; Gabrielli et al., 2011; Kunz et al., 2017; Autran et al., 2018), higher HMO content in preterm milk vs. term milk (Coppa et al., 1997; Sundekilde et al., 2016), especially the fucosylated ones (fucose, *N*-acetyl-neuraminic and *N*-acetyl-glucosamine) (Perrone et al., 2019), and more highly variable HMO composition in preterm milk (Nakhla et al., 1999; De Leoz et al., 2012). However, fucosylation of HMOs may not be as well-regulated in preterm milk as in term milk (De Leoz et al., 2012). A recent study described the dynamical evolution of HMO content of preterm and term milks in 53 mothers over the first four and the first two postnatal months of lactation, respectively (Austin et al., 2019) (Figure 1). Although the concentration of most HMOs was comparable at equivalent postpartum age, that of 3-sialyllactose (3′SL), DSLNT and Siallylacto-*N*-neo-tetraose b (LSTb) was higher and that of 6-sialyllactose (6′SL) and Siallylacto-*N*-neo-tetraose c (LSTc) was lower in preterm than term milk. Furthermore, lacto-*N*-neotetraose concentration was shown to significantly decrease in term milk over time but did not significantly change in preterm milk (Spevacek et al., 2015). Interestingly, however, a study documenting the changes in metabolomic profile of preterm and term milks highlighted that the preterm milk metabolome changed within 5–7 weeks postpartum to resemble that of full-term milk (Sundekilde et al., 2016). The effect of HMO content on neonatal microbiota agrees with a highly selective prebiotic effect of HMOs that shape the gut microbiota in the first weeks of life (Spevacek et al., 2015). Indeed, the primo-colonization of the intestine with bacterial populations composed primarily of non-HMO-consuming *Enterobacteriaceae* and *Staphylococcaceae* resulted in an increase of fecal HMOs when the further higher proportion of HMO-consuming *Bacteroidaceae* and *Bifidobacteriaceae* was associated with a reduction in fecal HMOs (De Leoz et al., 2015).

#### Bacteria and Fungi Content in Preterm and Term Breast Milk

The discovery of a HM microbiota, from the 2000s, has led many teams to question its origin and its relative role, compared to other microbiota (particularly maternal fecal microbiota), in the gut colonization of the newborn. HM guarantees a constant supply of bacteria throughout the lactation period, values ranging from 10^2^ to 10^4^ bacterial cells/mL with a culture-dependent analysis and around 10^6^ bacterial cells/mL with a qPCR-based analysis (Jost et al., 2013; Boix-Amorós et al., 2019). As reported in extensive reviews (Fitzstevens et al., 2017; Oikonomou et al., 2020), HM microbiota is a diverse and complex community with *Streptococcus* and *Staphylococcus* as the dominant genera in most of the studies using either culture-independent or culture-dependent approaches. *Propionibacterium*, *Bifidobacterium*, *Bacteroides*, *Enterococcus*, *Lactobacillus*, *Acinetobacter*, and *Veillonella* are, among others, the most cited dominant taxa in HM. Strong inter-individual variations exist that may be explained by several host and environmental factors including maternal body mass index, diet, time of lactation, ethnicity as well as geographical location (Gomez-Gallego et al., 2016; Kumar et al., 2016). Only one study investigated the effects of stages of gestation on milk microbiota from 39 Caucasian Canadian women (Urbaniak et al., 2016). No significant difference in the microbial profiles of milk between preterm and term births was observed. More studies are still needed with larger samples sizes to better assess whether stages of gestation impact milk microbiota composition.

Only limited data are available on the quantitative and qualitative fungal load of human breast milk compared to animal milks. Using multiple approaches, from culture to molecular techniques, Boix-Amorós et al. (2017) showed that 89% of samples had detectable levels of fungal DNA (median load of 3.5 × 10^5^ cells/ml). The three more abundant yeast genera found were *Malassezia, Candida*, and *Saccharomyces*. At the species level, yeast species detected by pyrosequencing and/or culture were both typical yeasts from the skin and/or oral cavity, such as *Malassezia globosa, Malassezia restricta, Candida parapsilosis, Candida albicans, Yarrowia lipolytica, Saccharomyces cerevisiae*, or *Rhodotorula mucilaginosa*. Filamentous fungi were also detected, and the most prevalent were *Alternaria* and *Cladosporium* species. More recently, a high-throughput approach using next generation sequencing allowed to characterize the human milk mycobiome from various countries and continents (Boix-Amorós et al., 2019). Basidiomycota (58.65%) and Ascomycota (41.03%) account for the most important phyla. *Malassezia, Davidiella, Sistotrema*, and *Penicillium* were the four more abundant genera detected.

### Pasteurized Human Donor Milk

The World Health Organization recommends pasteurized human donor milk for preterm infants when the mother’s own milk is unavailable (World Health Organization, 2011). In preterm and low birth weight infants, evidences may indicate that feeding with human donor milk, either as a supplement to maternal expressed breast milk or as a sole diet resulted in a lower risk of developing NEC compared with formula (Quigley et al., 2019). Although human donor milk feeding was associated with lower rates of weight gain, growth, and head growth, no effect of milk feeding types on all-cause mortality, long-term growth and neurodevelopment has been observed. The inactivation of milk bacteria and the significant reduction of bioactive proteins and enzymes resulting from the pasteurization process of the human donor milk may limit some of its health benefit compared to a mother’s own milk. Indeed, bioactive components such as Igs, lactoferrin, enzymes (lipase), HMOs and vitamins are reduced or inactivated after pasteurization. In addition, pasteurization associated to freeze-thaw cycles may modify the kinetics of digestion of proteins and lipids by selectively modulating the intestinal release of amino acids and decreasing that of some fatty acids as demonstrated using an *in vitro* dynamic digestion system (de Oliveira et al., 2016). However, it is noticeable that pasteurized HM favored an intestinal microbiome in infants more similar to mother’s own milk than formula despite the differences in bacterial content between raw and pasteurized breast milks (Parra-Llorca et al., 2018). All these data suggests that the pasteurization of HM preserves most of functionalities of breast milk in terms of influence on the intestinal microbiota profile and health outcomes in preterm infants.

### Fortified Milk for Preterms

Evidence indicates that HM is the best nutritional standard for infants that suits not only to term but also to preterm infants, especially those born with a very low birth weight (VLBW), conferring both short and long-term health benefits (Tudehope, 2013). However, HM does not meet the higher nutritional requirements of preterm and VLBW infants compared to those of term infants (Ballard and Morrow, 2013; Simpson et al., 2016) when fed at the usual feeding volumes during the first weeks of life. This may lead to postnatal growth restriction with the associated risk of impaired neurodevelopment and other poor health outcomes (Chan et al., 2016; Guellec et al., 2016; Coviello et al., 2018). Nutrient requirement of preterm infants is defined as intakes that enable the neonate to grow at the same rate as a fetus (Ziegler, 2014). Any shortfall in protein supply affects early growth of preterm infants and increases the risk for inadequate growth during later childhood and adulthood (van Beek et al., 2020). Thus, protein supply needs special attention in early life with the aim to meet the protein requirement of growing preterm infants ranging between 3.5 and 4.5 g/kg/day (Agostoni et al., 2010; Ziegler, 2014). However, feeding with 150 mL/kg/day of unfortified HM (often considered full enteral feeding) provides only about 1.8 g/kg/day of protein. Optimal early nutrition of preterm infants is thus facilitated by using multi-nutrients human milk fortifier (MF) that increases the concentration of nutrients to their requirement levels at the recommended feeding volumes (135–200 ml/kg/d) (Ziegler, 2011). HM fortification is now commonly recommended in neonatal intensive care units (NICU) of very preterm infants with a birthweight <1,800 g (Moro et al., 2015). HM should be fortified with the nutrients in short supply, particularly with proteins, minerals such as calcium and phosphate, as well as micronutrients such as iron, zinc, copper, selenium and iodine (Arslanoglu et al., 2019). MF can be used safely as soon as the milk volume reaches 50–80 ml/kg/d. In contrast, no strong evidence supports the use of hydrolyzed protein source in MF (Arslanoglu et al., 2019). MF are commonly derived from bovine milk. In fact, fortification of breast milk feeds with human MF in comparison with bovine MF did not reduce the risk of necrotizing enterocolitis in preterm infants (Premkumar et al., 2019). Moreover, current data do not provide guidance on the optimal time to start fortification (Alyahya et al., 2020). Among the different fortification approaches, while “Standard fortification” falls short in supplying sufficient proteins, current recent data encouraged the use of “Individualized Fortification” (adjustable and targeted fortification) to optimize nutrient intake (Arslanoglu et al., 2019).

### Impact of Maternal Nutrition on Human Milk Quality

Maternal nutrition may influence breast milk composition in mothers who delivered prematurely (Hascoët et al., 2019). However, only overall maternal carbohydrate intake was positively correlated with milk protein, fat and caloric density as observed in a French cohort of 81 mothers who delivered prematurely (between 24 and 34 weeks’ gestational age), regardless of the kind of carbohydrate: sugar, fibers, or overall carbohydrates (Hascoët et al., 2019). The absence of effects of overall protein intake on the milk composition corroborated findings in mothers who delivered at term, such as the study by Zhao et al. (1989) in two Chinese cohorts from 2 areas of China differing in the mothers’ overall protein intake, where no differences in 18 studied amino-acids could be find in breast milks. However, in a recent study with 220 Chinese lactating women who delivered at term, dietary patterns with high intake of red meat, cereals and eggs was associated with higher protein, total dry matter and energy content in HM (Huang and Hu, 2020). Regarding lipids, supplementation of maternal diet with the *n*−3 PUFA precursor ALA did not modify the milk overall lipid concentration but qualitatively increased the proportion of ALA, still in mothers who delivered at term (Mazurier et al., 2017). Similarly, Chilean women who had a low intake of foods that are natural sources of *n*−3 PUFAs (vegetable oils, fish, and seafood) and a high intake of n−6 PUFAs (LA and AA) during lactation displayed a significant reduction of DHA levels in breast milk (Barrera et al., 2018). Interestingly, the concentration of *n*−3 PUFAs in HM would be related to the mothers’ habitual but not current intake (Bzikowska-Jura et al., 2019), suggesting that current post-partum *n*−3 PUFA intake does not translate directly into their concentration in HM but is rather influenced by the maternal body stocks of FA. Pre-pregnancy obesity was associated with increased fat and energy content in HM 6 weeks after preterm delivery, which may be due to higher blood triglycerides in the obese or to oxidative stress and inflammation caused by obesity, with consequences for HM metabolomic profile (Burianova et al., 2019). A negative correlation between mother’s weight and cholesterol concentration in HM has been described (Kamelska et al., 2012). The dietary intake of polyamines was significantly associated with the polyamine content of transitional HM collected in mothers who delivered prematurely, especially for spermidine and putrescine (Atiya Ali et al., 2014). Furthermore, total polyamine level was higher in preterm milk, with the levels of putrescine and spermidine being 50 and 25% higher, than in term milk (Plaza-Zamora et al., 2013; Atiya Ali et al., 2014). The impact of maternal diet on HMO content has not been clearly addressed so far. Azad et al. (2018) found no association between overall diet quality and HMO concentration. However, maternal probiotic supplementation during late stages of pregnancy was recently associated with change in breast milk HMO composition (Seppo et al., 2019). Moreover HMO diversity and the concentration of LNnT were negatively correlated to prepregnancy body mass index, whereas 2′FL was positively associated to prepregnancy body mass index in secretor mothers, in a Finish cohort including both term and preterm-delivered women (Lagström et al., 2020). HM from obese women displayed increased levels of monosaccharides and sugar alcohols including mannose, ribose, lyxose, lyxitol (Saben et al., 2020) and Berger et al. (2018) revealed that mothers who consumed a beverage sweetened with high-fructose corn syrup had increased fructose in HM.

In a recent study, which specifically addressed the effect of maternal nutrition on HM microbiome, Babakobi et al. (2020) found a significant negative correlation between *Streptococcus* relative abundance in HM and maternal intake of PUFAs and folic acid, as well as with HM oleic acid content. Additional correlations were detected between *Staphylococcus hominis* and two MCFAs (C8:0 and C12:0) (Babakobi et al., 2020).

## Gut Microbiota in Preterm Infants and Impact of Environmental Factors

### Initial Bacterial and Fungal Colonization: Preterm vs. Term Infants

Recent articles showed bacterial presence in the amniotic fluid, umbilical cord and the placenta (Wassenaar and Panigrahi, 2014). While these observations suggest that colonization of the fetal gut may begin *in utero*, several other studies have put forward arguments against such a possibility (Salter et al., 2014; Lauder et al., 2016; Perez-Muñoz et al., 2017). The first meconium is rich in genera such as *Escherichia-Shigella*, *Enterococcus*, *Leuconostoc*, *Lactococcus*, and *Streptococcus* (Gomez-Gallego et al., 2016). Initial colonizing fungal species are also present in 71% of infants and include *C. albicans* and *C. parapsilosis* (Kaufman et al., 2006). At birth, the infant gut is an aerobic environment, which gradually becomes anaerobic over a period of days (Johnson and Versalovic, 2012; Jost et al., 2012). The meconium facultative aerobic bacteria that firstly colonize the infant gut (*Escherichia* and *Enterococcus*) eventually establish an anaerobic environment, and promote the colonization with obligate anaerobes, including Firmicutes such as Clostridia, Bacteroidetes, and especially the protective Bifidobacteria (Mackie et al., 1999). Bifidobacteria comprise the largest group within the infant microbiota (Jost et al., 2012; Jakobsson et al., 2014). Throughout this succession of organisms, the microbiota increases in diversity (Koenig et al., 2011; Jakobsson et al., 2014). At the opposite, gastrointestinal fungi are present at a significantly higher diversity during the first 3 months of life than later in life (Kaufman et al., 2006).

The microbiota development of infant gut is dependent on gestational age at time of delivery and, for term infants, mode of delivery (Dominguez-Bello et al., 2010; Makino et al., 2013; La Rosa et al., 2014). Term infants born vaginally are initially colonized by microbial communities resembling maternal vaginal microbiota (*Lactobacillus*, *Prevotella*, *Candida*, *Davidiella* and *Cladosporium*). In contrast, fecal microbiota of term infants delivered by cesarean section more closely resembles the skin microbiota (*Staphylococcus*, *Propionibacterium*, and *Malassezia*) (Dominguez-Bello et al., 2010; Nagata et al., 2012; Drell et al., 2013). Contrarily, the mode of delivery does not appear to significantly affect the initial colonizing community in preterm infants, which instead is hypothesized to be highly influenced by the environment (Schwiertz et al., 2003; Brooks et al., 2014). For preterm infants (gestational age <33 weeks) the early gut microbiota composition resembles bacterial communities colonizing hospital surfaces and feeding and intubation tubing and are enriched in *Staphylococcus epidermis*, *Klebsiella pneumoniae*, and *Escherichia coli* (Brooks et al., 2014). Preterm infants were also shown to be colonized early with *C. albicans* for a median range of 3 weeks and *C. parapsilosis* for 4 weeks (Kaufman et al., 2006). The examination of the gut-associated microbiota of 11 extremely low birth weight preterm infants in the first postnatal month revealed that the most prevalent and abundant fungi were *Saccharomyces* (*S. cerevisiae*) followed by *Candida* (*C. albicans*, *Candida glabrata*, *C. parapsilosis*, *Candida tropicalis*, *Candida Diddensiae*, and *Candida quercitrusa*) (LaTuga et al., 2011). The susceptibility to *Candida* invasive infection has been correlated with a naïve immune system, a bacterial dysbiosis and use of parenteral nutrition (Kumar et al., 2016).

**FIGURE 2:**
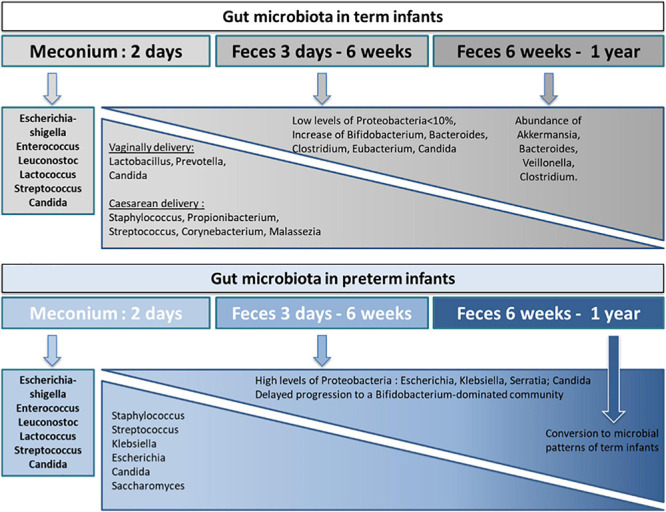
Key bacterial and fungal colonization of the term and preterm infant gut microbiota from birth through 1 year of life. The composition of the gut microbiota in term and preterm infants varies differently from birth before reaching an identical composition at the first year of life. During the first year of life, pro-inflammatory colonizers are more present in preterm infants than in term infants. The developing term infant gut microbiota is initially dominated by Firmicutes, with low levels of Proteobacteria species, following by an increase in *Bifidobacterium* and *Bacteroides* within the first 6 months of life. In contrast, preterm infant gut microbiota is quickly dominated by Proteobacteria species within the first week of life, which remain at high levels throughout the first month and thus induce a delayed progression to a *Bifidobacterium*-dominated community compared to term infants. In the case of a vaginal delivery, early colonizers originate from the mother’s vaginal and fecal microbiota whereas for C-sections, early colonizers belong to the environment of birth and the mother’s skin microbiota. The gut microbiota differences between vaginally and caesarean delivery disappear after the first year of life.

Following initial colonization, gut microbiota of both term and preterm infants increases in diversity and dynamically changes in composition. However, specific bacterial succession patterns are unique to these two populations (Schwiertz et al., 2003; Yatsunenko et al., 2012; La Rosa et al., 2014). The most notable difference concerns the enrichment in Proteobacteria before 2 weeks of age in preterms. The developing term infant gut microbiota is initially dominated by Firmicutes, when Proteobacteria species are sparsely present in the first week of life and persist as minor components (<10% relative abundance on average) throughout the first 2.5 years of life (Koenig et al., 2011). Thereafter there is an increase in *Bifidobacterium* and *Bacteroides* in healthy term infants within the first 6 months of life (Jost et al., 2012; La Rosa et al., 2014; Korpela et al., 2018). In contrast, preterm infant gut microbiota is quickly dominated by Proteobacteria (facultative anaerobes) species within the first week of life, which remain at high levels (>75% relative abundance on average) throughout the first month (Morowitz et al., 2011; La Rosa et al., 2014). Because of a prolonged dominance of Proteobacteria such as Enterobacteriaceae, preterm infants have a delayed progression to a *Bifidobacterium*-dominated (obligate anaerobes) microbiota compared to term infants (Butel et al., 2007; Bäckhed et al., 2015; Korpela et al., 2018). Overall, the intestinal microbiota from preterm infants up to 5 weeks of age clusters distinctly from that of full-term breastfed infants and the microbial patterns converge toward that of full-term breastfed infants only at or after 6 weeks of age (Claud et al., 2013). By the end of the first year of life, the infant gut microbiota begins to resemble an adult-like microbiota, reaching full maturity by 2–3 years of age (Palmer et al., 2007; Koenig et al., 2011; Yatsunenko et al., 2012). At 1 year of age, the infant gut microbiota has a characteristic abundance of *Akkermansia muciniphila*, *Bacteroides*, *Veillonella*, *Clostridium coccoides*, and *Clostridium botulinum* (Alou et al., 2016) and is dominated by three bacterial phyla: Firmicutes (*Lachnospiraceae* and *Ruminococcaceae*), Bacteroidetes (*Bacteroidaceae*, *Prevotellaceae*, and *Rikenellaceae*), and Actinobacteria (*Bifidobacteriaceae* and *Coriobacteriaceae*). These data demonstrate that while the succession of bacteria at the genus level is similar, dominance is different between preterm and term infants in the early period of life (Figure 2).

It is noteworthy that the severity of prematurity seems to be a major driver in the development of the microbiota for preterm infants, specifically the slowest rate of assembly was found in the most premature infants (between 25 and 30 weeks of gestational age) (La Rosa et al., 2014; Dahl et al., 2018; Korpela et al., 2018). The preterm infant microbiota may follow an age-dependent maturation which may be slightly dependent on a specific gestational age (Claud et al., 2013). The microbial community differences between preterm and term infants are due to differences in the environment of NICU as above-mentioned, but also to an immaturity in the intestinal epithelium (Claud et al., 2013).

### Bacterial and Fungal Profiles of Preterm Infant Feces Associated With Breast Milk and Formula Feeding

Whether the term infants are breastfed or formula-fed affects their gut microbiota (O’Sullivan et al., 2015; Boudry et al., 2021). Breast-fed term infants generally become colonized in the first weeks after birth with the protective bacterial *Bifidobacterium* and *Lactobacillus* that are able to consume HMOs whereas formula-fed infants have a higher diversity and levels of *Enterobacteria*, *Clostridia*, *Bacteroides*, *Enterococcus*, and *Streptococcus* (Favier et al., 2002; Penders et al., 2006; Bezirtzoglou et al., 2011; Azad et al., 2013; Bäckhed et al., 2015). Even if formula-fed infant harbor *Bifidobacterium* in their gut microbiota, breastfed infant gut is generally colonized with a more complex and diverse *Bifidobacterium* ecosystem and a twofold higher number of *Bifidobacterium* cells compared to formula-fed infants (Roger et al., 2010; Bezirtzoglou et al., 2011). In preterm infants, the impact of HM feeding on infant gut microbiota is not clearly assess due to the low numbers of preterm infants exclusively breastfed and the various timings in which formula feeding is introduced exclusively or in supplementation observed in clinical studies (Dahl et al., 2018). Indeed, preterm infant gut microbiota is enriched in microbes that commonly dominate in the presence of antibiotics (Wandro et al., 2018). However, vaginally born, exclusively breastfed preterm infants not exposed to antibiotics had fewer Firmicutes and more Proteobacteria than children born at term (Dahl et al., 2018). Preterm infants fed infant formula had a lower initial bacterial diversity and a less gradual increase in diversity compared to preterm infants who were fed HM (Gregory et al., 2016). Furthermore, the microbiota of preterm infants fed HM clusters regardless of birth weight, when that of preterm infants fed infant formula clustered differently based on birth weight (Gregory et al., 2016). The ordered succession of microbial taxa observed in HM-fed preterm infants was disrupted in those fed infant formula (Gregory et al., 2016). Fecal microbiota of preterm infants fed with exclusive own mother’s HM presented increased richness compared to those fed with different proportions of formula. In addition differences in microbiota composition were reported: the mean proportion of *Escherichia* and *Clostridium* was always greater in preterm infants who received diets containing formula than in preterm infants fed with HM only (Zanella et al., 2019). It was also shown in a cohort of 3,161 preterm infants that a slow rate of progression of enteral feeding and a less favorable direct-breastfeeding policy was associated with colonization by *Clostridium neonatale* and/or *Staphylococcus aureus* and most likely with the increased risk of developing NEC (Rozé et al., 2017). The type of diet of the infant (formula-fed or breast-fed) does not seem to impact oral fungal profile, although fungi are present in HM (Boix-Amorós et al., 2019).

### Antibiotic, Antifungal, and Disinfectant Exposure (Environmental Factors) and Microbial Profiles

#### Antibiotics

The composition of gut microbiota can be affected by the timing, duration, and type of antibiotic exposure (class, dose, period of exposure, pharmacological action, and targeted bacteria) in both preterm and term infants (Fouhy et al., 2012; Gasparrini et al., 2016; Gibson et al., 2016; Yassour et al., 2016; Iizumi et al., 2017). Specific properties of antibiotics such as antimicrobial effects or mode of action select intestinal bacteria and induce shifts in bacterial composition during antibiotic therapy (Pérez-Cobas et al., 2013). Intrapartum antibiotic prophylaxis induced lower gut diversity and abundance of *Lactobacillus* and *Bifidobacterium* in neonates (Mueller et al., 2015).

In preterm infants, meropenem, cefotaxime, and ticarcillin–clavulanate are associated with significantly reduced species richness. In contrast, vancomycin and gentamicin, the antibiotics most commonly administered to preterm infants, have non-uniform effects on species richness (Gibson et al., 2016). In term infants, parenteral ampicillin and gentamicin administration (within 48 h of birth) resulted in a significantly increased abundance of Proteobacteria and decreased abundance of Actinobacteria (particularly *Bifidobacterium*) and *Lactobacillus* 4 weeks after the treatment compared to the untreated controls (Fouhy et al., 2012).

#### Antifungals

Preterm or VLBW infants have been identified as a high risk group of patients to be colonized with fungi and further to develop invasive fungal infections (Manzoni et al., 2015). The high mortality of these infections mainly due to *Candida* sp. conducted to the implementation of a randomized clinical trial to evaluate the performance of prophylactic administration of fluconazole during the first 6 weeks of life (Kaufman et al., 2001). Results showed that prophylaxis with fluconazole was effective in decreasing fungal colonization and preventing invasive fungal infection in VLBW infants. It is noticeable that fluconazole prophylaxis did not induced non-*albicans* species selection nor emergence of fluconazole -resistant strains in a wide Italian study in preterm neonates in NICU over a 16-year surveillance period (Luparia et al., 2019) compared to hematological patients. However, despite the administration of antifungals, fungal colonization still occurred in 7 out of 11 extremely low birth weight infants (LaTuga et al., 2011). Although antibiotics do not directly act on fungi, anti-bacterial antibiotic exposure is associated with an increased rate of fungal colonization (Dollive et al., 2013). In preterm infants, exposure to cephalosporins is associated with an increased risk for invasive candidiasis (Kelly et al., 2015). The aerobic growth of *C. albicans* has been studied extensively. Interestingly the facultative anaerobic growth has been showed *in vitro* (Dumitru et al., 2004). This anaerobic growth of *C. albicans* may contribute to the resistance of *C. albicans* biofilms to antifungal drugs.

#### Disinfectant

A strong link has been shown between NICU-specific taxa and their presence in the gut microbiota of preterm infants, mostly mediated by healthcare providers and cleaning protocols (Brooks et al., 2018). Therefore, approaches that aim to change NICU microbiota may be an effective way to manipulate the early microbiota of preterm infants.

To summarize, the early window for gut microbiota establishment is critical. Gestational age, mode of delivery as well as environmental factors (antibiotic, antifungal, and disinfectant) largely affect gut microbiota establishment. However, nutrition also shapes the assembly of infant gut microbiota with significant functional implications (Fragkou et al., 2021).

## Dysbiosis, Brain Development and Neurodevelopmental Outcomes

### Post-natal Development of the Brain

The brain undergoes a rapid trajectory of growth during the third trimester of gestation, with a 140% volume increase from the 30th to the 40th week of gestation, resulting in a neonatal brain representing about 36% of the adult volume at full-term birth. Brain structure and function continue to mature during the early post-natal life to reach 80–90% of the adult brain volume by the age of 2 (Knickmeyer et al., 2008). During this period, dendrites, axons, new synapses and glia cells expand, and myelination occurs. During the first two postnatal years, synapse production occurs with a peak between 3 and 24 months depending on the cortical region (Huttenlocher and Dabholkar, 1997). Synaptic refinement and elimination as well as myelination continue in late childhood and beyond adolescence (Petanjek et al., 2008). Prefrontal regions are among the last brain areas to reach mature levels (Giedd, 2004). Interestingly, magnetic resonance imaging (MRI) studies reveal that despite normal head circumference at birth, autistic children display abnormal regulation of brain growth resulting from early overgrowth followed by abnormally slowed growth (Courchesne et al., 2001; Hazlett et al., 2005) highlighting that adequate, i.e., not too slow but not too rapid, growth is a key parameter for later brain health.

In preterm infants, brain growth is a major matter of concern. Larger total brain tissue, white matter, and cerebellar volumes at term-equivalent age are associated with better neurodevelopment in very, moderate or late preterm children (Cheong et al., 2016; Schneider et al., 2018). Impaired neuronal connectivity, likely associated with impaired dendritic growth and synapse formation, was also observed in preterm infants during infancy and beyond, correlating with lower neurodevelopmental scores in adolescence (Constable et al., 2008; Dean et al., 2014). Despite intensive research and refinement of methods, the mechanisms leading to normal brain growth and maturation during the post-natal life are still not fully understood, especially in preterm infants. The role of environmental factors such as nutrition has been described (Schneider and Garcia-Rodenas, 2017). However, microbiota is another potentially key actor that starts to emerge.

### Evidences for a Role of the Microbiota in Neonatal Brain Development

#### Evidences From Preclinical Models

The germ-free (GF) mouse model has been widely used as a first approach to demonstrate the role of microbiota on brain development and function. These mice display decreased anxiety-like behavior compared to conventional mice at adulthood and numerous alterations at the brain level (Luczynski et al., 2016). Indeed, compared to conventional mice, GF mice exhibit many brain alterations: higher expression of synaptic-related proteins (synaptophysin and PSD-95) in the striatum (Diaz Heijtz et al., 2011), increased levels of key myelin-associated genes and hypermyelination in the prefrontal cortex (Hoban et al., 2016), increased hippocampal cell survival (Ogbonnaya et al., 2015), reduced subventricular zone cell proliferation (Sawada et al., 2018), increased blood–brain-barrier permeability (Braniste et al., 2014), impaired microglia immune response and immature morphology (Erny et al., 2015), altered hippocampal microRNA and mRNA expression (Zhou et al., 2019), increased expression of splicing factors upon stimulation in the amygdala (Sawada et al., 2018; Stilling et al., 2018), decreased expression of the total brain-derived neurotrophic factor (BDNF) in the amygdala (Arentsen et al., 2015), and numerous alterations in neurotransmitter and receptor expression (Luczynski et al., 2016). Collectively, these data indicate a role of the microbiota on brain function, and especially on processes that are activated during brain post-natal maturation. Interestingly, most, but not all, of these alterations can be corrected upon colonization of GF mice at weaning. This both-ways reversible effect suggests that it may be possible to counterbalance such effects in certain conditions.

Beside GF models, early life intervention on gut microbiota in preclinical models also demonstrated long-term effect of neonatal dysbiosis on behavior. Indeed, oral administration of antibiotics (ampicillin or a cocktail of neomycin, bacitracin, and pirimacin) to pregnant mice leads to modification in motor activity of their offspring and altered social behavior, but only in the male offspring (Arentsen et al., 2017). Low-dose penicillin from late pregnancy until weaning decreased anxiety-like behavior in young adult male mice (Leclercq et al., 2017). Interestingly, concurrent supplementation with a probiotic strain (*Lactobacillus rhamnosus* JB-1) prevented some of these alterations (Leclercq et al., 2017). Moreover, neonatal antibiotic treatment (vancomycin) affected visceral pain in adulthood, but did not impact cognitive or anxiety-related behaviors in male rats (O’Mahony et al., 2014).

Overall, the impact of antibiotics on brain development and behavior seems to be highly dependent upon factors such as the type and the dose used and the developmental time window when such exposure(s) occurred.

#### Evidences From Human Data

##### Impact of factors affecting the natural microbiota assembly on neurodevelopment

The first evidences of the role of early microbiota composition or metabolic capacity and activity on neurodevelopment arise from studies linking factors that affect the natural assembly of the neonatal gut microbiota (mode of delivery, use of antibiotic, nutrition, etc.) and neurodevelopment. These studies should, however, be considered with caution, since these factors do not only affect gut colonization but also other pathways potentially affecting brain development. Nonetheless, these studies give a first idea of the link between microbiota and brain maturation in infancy.

##### Mode of delivery

C-section was associated with a delay in personal social skills and gross motor function at 9 months but not 3 years of age in a cohort of 11,000 infants (Al Khalaf et al., 2015). Likewise, a retrospective study with a lower number of infants (*n* = 104) but followed until 10 years of age indicated that C-section born infants have later attainment of developmental milestones compared to vaginally-born infants (Chojnacki et al., 2019). However, when studying brain health (attention deficit disorders and autism spectrum disorders), studies are very inconsistent and meta-analysis or large cohort studies could not conclude to any association (Curran et al., 2015, 2016).

##### Use of antibiotics

As antibiotics have long been considered safe, there is a paucity of studies evaluating the consequences of antibiotic treatment on brain development and subsequent function and behavior. A recent study on 342 children reported that antibiotic treatment in the first 6 months of life may trigger behavioral changes in children at 11 years of age, such as lower overall cognitive and verbal comprehension abilities, increased risk of problems with metacognition, executive function, impulsivity, attention-deficit hyperactivity, anxiety and emotion (Slykerman et al., 2019). This confirms a first study from the same research group in another cohort indicating that children that had received antibiotics in the first year of life had more behavioral difficulties and symptoms of depression at 11 years of age (Slykerman et al., 2017; Lavebratt et al., 2019). Moreover, it is noteworthy that drugs over than antibiotics can also have anti-microbial properties. The impact of early-life administration of these drugs on brain development warrants further investigations.

##### Nutrition

It has long been established that breast-feeding was associated with better neurodevelopment (Kramer et al., 2008; Quigley et al., 2012). Importantly, the effect is particularly noteworthy for preterm infants, who have an increased risk for behavioral problems and cognitive impairments later in life (Quigley et al., 2012). Recent data indicated that breast-feeding more than 1 month was associated with higher IQ compared to breast-feeding less than 1 month (Strøm et al., 2019). However, even though breast-feeding impacts gut microbiota composition, increasing for instance *Bifidobacterium* abundance, it is difficult to link these results to microbiota composition only as breast-milk provides specific nutrients known for their beneficial effects on brain development (LC-PUFAs, HMOs, etc.). However, *Bifidobacterium* species are responsible for the fermentation of HMOs to produce SCFAs (Marcobal et al., 2011). SCFAs improve the gut health by promoting intestinal barrier integrity maintenance, mucus production and intestinal hormone secretion (Gaudier et al., 2009; Peng et al., 2009; Tolhurst et al., 2012). In addition to exerting local effects in the colon, SCFAs play a pivotal role in microbiota-gut-brain crosstalk. Accumulating evidence suggests that SCFAs that cross the blood-brain barrier into the central nervous system have neuroactive properties. A multitude of animal studies have shown that SCFAs widespreadly may be involved in critical phases of neurodevelopmental and neurodegenerative disorders (Sharon et al., 2016; Dinan and Cryan, 2017; Fung et al., 2017; Kelly et al., 2017; Dalile et al., 2019). MFGM or some specific MFGM components such as sphingomyelin (phospholipids) have been reported to have positive association with the neurobehavioural development of infants born at term (Tanaka et al., 2013; Timby et al., 2014) and to change, though moderately, oral microbiome (lower level of *Moraxella catarrhalis* with MFGM supplementation compared to standard formula feeding) (Timby et al., 2017a). A recent systematic review on the effects of different nutritional interventions, including supplementation with amino acids and protein, lipids, probiotics, prebiotics, vitamins, and minerals, to reduce brain injury and/or improve neurodevelopmental outcomes in preterm infants (24 randomized controlled clinical trials included) concluded that positive effects of nutritional interventions have not been evidently demonstrated in these trials even though promising effects have been demonstrated in many pre-clinical studies (Hortensius et al., 2019). Concerning probiotics, ***Lactobacillus acidophilus*** and ***Bifidobacterium infantis*** (Chou et al., 2010), **a combination of *B. infantis, Streptococcus thermophilus*, and *Bifidobacterium lactis*** (Jacobs et al., 2017) **or *Lactobacillus Sporogenu*** (Sari et al., 2012), **were administered the first week after birth until discharge and no significant effect of supplementation on neurodevelopmental outcome assessed between 2 and 5 *years was found. It is noticeable that single, but not multiple, nutritional interventions were included in these studies. In addition, several relevant factors such as the type of infant nutrition (i.e., breast milk vs. formula or donor milk), the timing and the dose of the nutritional supplementation hampered the conclusions in preterm infant studies*** (Schneider and Garcia-Rodenas, 2017).

#### Observational Prospective Studies

##### Behavior and temperament

Few clinical studies evaluated the association between microbiota composition and later child behavior and temperament. A first exploratory investigation evaluated fecal microbiota composition (through 16S sequencing) and concomitant temperament, rated by maternal questionnaires, in full-term healthy toddlers aged 18–27 months. Phylogenic diversity was associated with higher surgency/extraversion scores in both boys and girls (Christian et al., 2015). However, this study did not evaluate microbiota during infancy, which is the most rapid period of neurodevelopment. A second prospective study evaluated the link between microbiota composition (also through 16S sequencing) at 2.5 months of age, i.e., during infancy, and temperament at 6 months in 300 full-term infants. Using a clustering strategy, they identified three distinct groups (discriminated by Bacteroidetes, *Veillonella dispar* and *Bifidobacterium*/Enterobacteriaceae, respectively). The Bacteroidetes cluster was associated with lower self-regulation capacity (a dimension that includes cuddliness, soothability, and orienting), compared to the *Bifidobacterium*/Enterobacteriaceae cluster. Moreover, negative emotionality and fear reactivity that may predict development of anxiety later in life, was associated with reduced diversity at the age of 6 months (Aatsinki et al., 2019). Finally, Loughman et al. (2020) used a different strategy to evaluate the link between infant behavior and microbiota: they looked for difference in microbiota composition at 1, 6, and 12 months of age (16S sequencing) of infants separated in behavior vs. non-behavior cases, based upon parent questionnaires completed at 2 years of age. While microbiota composition at 1 and 6 months of age was not different between the two populations, the presence of *Prevotella* at 12 months was significantly lower in the group of behavior cases (Loughman et al., 2020).

Overall, these studies point out the existence of correlations between microbial profiles and infant behavior but the identification of causal relationships warrants further investigations.

##### Cognitive development

Recently, two prospective observational studies linked early-life microbiota composition to cognitive neurodevelopmental scores in full-term infants. Carlson et al. (2018) analyzed fecal microbiota composition through 16S sequencing at 1 year of age in 89 full-term healthy infants and correlated them to the Mullen Scale of Early Learning (MSEL) scores and brain anatomy (MRI) at 1 and 2 years of age. Using a clustering strategy, they identified three groups of infants differing by their bacterial composition at 1 year of age [discriminated by *Faecalibacterium*, Bacteroidetes (and low Lachnospiraceae), and Ruminococcaceae, respectively]. These groups differed in term of MSEL cognitive score at 2, but not 1, years of age. Indeed, infants in the Bacteroidetes cluster performed better, especially in the receptive and expressive language dimensions, than those in the *Faecalibacterium* and Ruminococcaceae clusters. Interestingly, predictor covariate of this better-performing group was breast-feeding and vaginal birth. Unrelatedly to this clustering approach, the authors also observed negative correlation between alpha-diversity at 1 year (number of observed species, Chao1 index, Faith’s phylogenetic diversity) and MSEL cognitive score at 2 years. This would suggest that, counterintuitively, a too great microbiota diversity at this period of life might be detrimental to neurodevelopment that would need confirmation in other studies. Association between microbiota composition or alpha-diversity and neuroimaging data indicated minimal effects of microbiota at 1 year on brain volume at 2 years of age.

A more recent study, enrolling 309 full-term healthy infants, evaluated the relationships between fecal microbiota composition, also estimated through 16S sequencing, at 3–6 months of age and score of the Age and Stage Questionnaire (ASQ) at 3 years of age (Sordillo et al., 2019). The authors used a co-abundance factor approach, which allowed assigning four scores to each individual based on the co-abundance of the 25 most abundant bacterial taxa. They then mathematically correlated these microbiota scores to the ASQ scores. Interestingly, scores in communication and personal social skills were negatively associated with the microbiota factor comprising relative high abundance of Lachnospiraceae and Clostridiales and low abundance of Bacteroidetes, while fine motor skills scores were negatively correlated with the factor comprising relative high abundance of Bacteroidetes and low abundance of *E. coli* and *Bifidobacterium*, two early colonizers. A tendency for increased Shannon diversity index with lower personal and social skills was also noticed.

These two prospective observational studies both pointed early relative abundance of Bacteroidetes and Lachnospiraceae as phylum/family correlating to later cognitive scores: high Bacteroidetes and low Lachnospiraceae relative abundances correlated with better receptive and expressive language scores and communication and personal and social skills, but lower fine motor scores for Bacteroidetes abundance. Both studies also pointed out the importance of low diversity for adequate neurodevelopment. However, these recent exciting data need to be confirmed by other studies focusing on microbiota composition during the early weeks postnatally.

A paradigm shift in the concept of the origin of human neurodevelopmental and psychiatric disorders has emerged since the discovery of the link between gut microbiota and brain function and behavior (Mayer et al., 2014). This link between early life gut colonization and later neurodevelopmental outcomes is starting to arise, demonstrated by pre-clinical interventional studies but also rare clinical studies, focusing on normal-weight healthy term babies. However, the role of gut colonization in preterm neonates, who constitute a high-risk population in term of neurodevelopment has been poorly explored so far. Moreover, assessment of fecal microbiota composition at a single time does not fully capture the early life dynamical changes in the microbiota, which may also be important to neurocognitive outcomes. Bioinformatical tools and models aimed at linking microbiota composition and neurodevelopmental questionnaires or behavioral traits are also needed to step forward in the understanding of the microbiota-brain axis. Finally, although in the aforementioned clinical studies, the effects are often small at individual levels and do not demonstrate an increased risk of clinically significant behavioral or cognitive problems. At a population level, these effects could have a more substantial impact on the prevalence of abnormality, particularly for the more high-risk population in term of neurodevelopment.

## Promising Microbiota-Modulation-Based Interventions for Neurodevelopmental Outcomes

Early body weight gain and macronutrient intake are positively related to brain volume and maturation and to neurodevelopmental outcomes in late infancy in very preterm infants (Coviello et al., 2018). As above reviewed several nutritional components of HM and infant formulas may influence gut microbiota which may have an impact on brain development and plasticity. Gut microbiota is capable to communicate with the central nervous system via the vagal nerve as well as microbiota-regulated intestinal production of cytokines, neurotransmitters, hormones and metabolites. Therefore, the development of microbiota modulation-based nutritional interventions may represent a novel strategy favoring gut colonization by beneficial bacteria and healthy microbiota with the aim of improving neurodevelopmental outcomes in preterm infants.

### Probiotics/Prebiotics

It is well established that there is a relationship between the bacterial communities in HM and those of the recipient infant’s feces (Lackey et al., 2019). Promoting breastfeeding is therefore an optimal way to ensure beneficial HM bacteria supply to the infant. However, when it is not wanted or possible, probiotic or prebiotic strategies may help to improve infant gut microbiota.

Prebiotics and probiotics are the most common ways to positively influence gut microbiota development in the early life. Prebiotics are defined as compounds that result in the ‘*selective stimulation of growth and activity of one or more microbial genus or species in the gut microbiota that confer health benefits to the host*’ (Roberfroid et al., 2010). The World Health Organization defines probiotics as ‘*live microorganisms which when administered in adequate amounts confer a health benefit on the host’* (Food and Agriculture Organization of the United Nations and World Health Organization, 2001). Without probiotic and prebiotic supplementation, the gut microbiota of formula-fed infants is generally not dominated by the Bifidobacterium species (Bezirtzoglou et al., 2011; Oozeer et al., 2013; Musilova et al., 2014).

Among *Lactobacillus* and *Bifidobacterium* species isolated from human milk, many of them (*Lactobacillus salivarius*, *Lactobacillus gasseri*, *Lactobacillus reuteri*, *Lactobacillus plantarum*, *Lactobacillus rhamnosus*, *Lactobacillus fermentum*, *Bifidobacterium breve*, *Bifidobacterium longum*, and *Bifidobacterium bifidum*) were included among the potentially probiotic ones and enjoyed the GRAS (Generally Recognized As Safe; FDA, United States) and the QPS (Qualified Presumption of Safety; EFSA, EU) status (Koutsoumanis et al., 2020).

#### Probiotic Therapy During Pregnancy or for Infant Nutrition

The first bacteria that colonize the infant gut at birth may originate from the maternal fecal, vaginal and breast milk microbiota, suggesting that the development of strategies to modulate maternal microbiota during pregnancy may be beneficial for neonates. In this context, *L. rhamnosus*, supplied to women during and after pregnancy, was correlated with an increase in the abundance of *Bifidobacterium* and *Lactobacillus* in the infant microbiota gut (Gueimonde et al., 2006; Lahtinen et al., 2009). In preterm infants, *Saccharomyces boulardii* can be used to regulate the growth of *Candida*. The enteral administration of bacterial and fungal probiotics, such as *L. reuteri*, *Lactobacillus casei*, *L. rhamnosus*, *L. acidophilus*, *S. thermophilus*, *Bifidobacterium longum*, *Bifidobacterium bifidum*, *Bifidobacterium lactis* and *S. boulardii* has also been used to reduce invasive candidiasis (Kaufman et al., 2006; Manzoni et al., 2015). In very preterm infants oral administration of either *L. rhamnosus* ATCC 53103 or *L. reuteri* ATCC 55730 was effective in the prevention of gastrointestinal colonization by *Candida* and may help to prevent suboptimal neurological outcomes (Romeo et al., 2011).

Several evidences from pre-clinical models clearly demonstrated the early interaction between microbiota and the brain and how probiotic supplementation may favor brain restoration, even though it was not clearly evidenced in human studies. The term ‘*psychobiotics to describe an emerging class of probiotics of relevance to psychiatry*’ has even been proposed (Dinan et al., 2013). Psychobiotics are probiotic strains that have shown behavioral effects in preclinical models, are able to promote the production of neuroactive substances such as gamma-aminobutyric acid and serotonin involved in the gut-brain axis, and have a capacity to decrease proinflammatory cytokines and reduce the hypothalamic–pituitary–adrenal (HPA) activity. Further research is warranted to expand the investigation of the benefits of psychobiotics within human preterm population as having a significant impact on cognition (Lv et al., 2021).

#### Microbiota Regulation Through Prebiotics

In pregnant women, galacto-oligosaccharides (GOS)/fructo-oligosaccharides (FOS) supplementation significantly increased fecal bifidobacterial levels, with potential benefits for the transmission of beneficial bacteria to their infant during the birth process (Firmansyah et al., 2016; Lv et al., 2021). The main prebiotics used in infant formula are short-chain GOS, long-chain FOS and polydextrose (PDX). Addition of GOS, a GOS/FOS mixture or PDX to infant formula has been shown to be effective in increasing lactobacilli and on bifidobacteria abundance (Knol et al., 2005; Rinne et al., 2005; Ben et al., 2008; Scalabrin et al., 2012; Sierra et al., 2015; Vandenplas et al., 2015). Finally, preterm infants given FOS-supplemented formula displayed an increased count of bifidobacteria and a corresponding significant reduction of *E. coli* and enterococci in fecal samples (Kapiki et al., 2007).

The use of prebiotics in infant formula is already a common practice to reduce digestive discomfort in term infants, even though scientific-based evidences are still too weak to state with certainty. In preterms, effects of GOS/FOS/pectin-derived acidic oligosaccharides on neurodevelopmental outcomes were measured by Bayley Scales of Infant and Toddler Development at 2 years (van den Berg et al., 2016). No significant improvement of neurodevelopmental outcomes was observed. However, lower bifidobacteria counts and higher serum cytokine levels during the neonatal period were associated with lower neurodevelopmental outcomes at 24 months of age, suggesting that an opportunity to influence neurodevelopment of these preterm infants via cytokine and microbiome modulation may exist (van den Berg et al., 2016). Future research is needed to provide more insights into the mechanisms of prebiotics and of combination of probiotics and prebiotics called ‘synbiotics,’ and their further use in preterm infants.

### Maternal Nutrition

As seen above, maternal nutrition is able to modulate, yet modestly, HM composition. One exciting strategy would be to try to modulate HM components known to drive infant gut microbiota, such as HM microbiota or HMOs or specific lipids content, through maternal nutrition but this remains very hypothetical to date. Indeed, the effects of maternal nutrition on infant microbiota have been poorly explored and deserve more studies. A recent study suggested that maternal diet does influence the infant gut microbiota and that these effects differ by delivery mode (Lundgren et al., 2018). Some effects of maternal diet were more apparent in exclusively breastfed infants, suggesting a role of HM in the effects observed on gut microbiota (Lundgren et al., 2018). Increased fruit intake by mothers was associated with increased belonging to the high *Streptococcus*/*Clostridium* group among infants born vaginally and maternal dairy intake was associated with increased belonging to the high *Clostridium* cluster in infant born by cesarean section (Lundgren et al., 2018). Some consistent associations between maternal DHA and EPA intake (through fish and seafood consumption) and infant gut microbiota profiles were also observed: maternal fish and seafood consumption was positively related to *Streptococcus* in the infant gut, and associated to a decrease in *Clostridium neonatale* in infants born by cesarean section (Lundgren et al., 2018). More indirectly, maternal gestational weight gain was associated with the infant fecal microbiota profiles (Robinson et al., 2017). Infant whose mothers had higher gestational gain weight were less likely to have a *Bacteroides*-dominant profile, had a lower bacterial community richness and Shannon diversity index (Robinson et al., 2017).

## Conclusion

Initial colonization and microbiota development in preterm infants differ from that in term infants. Increasing evidences suggest that intestinal dysbiosis in preterm infants predisposes the neonate to adverse neurological outcomes later in life. The increase in brain growth occurring in parallel with the infant microbiota complexification during the early period of life is a period of opportunity in which nutritional interventions (e.g., probiotics, psychobiotics, prebiotics, synbiotics, and maternal nutrition) may have their maximal effects on the gut-brain communication and provide benefits to enhance maturation and shape brain development. Consequently, it is essential to develop novel microbiota modulation-based nutritional interventions for infants and young children at high-risk for neurodevelopmental disorders. With the key characters of immaturity of preterm infants, we propose the possibility to manipulate the microbiota in early life as a preventive strategy to neurodevelopmental disorders. Identified critical windows may concern maternal nutrition through pregnancy and lactation and the infant nutrition during the early postnatal period (0–6 weeks of life). Translational research in this field is limited and further investigation of the efficacy of nutritional strategies in early life is warranted.

## Author Contributions

SB-B, GB, SB, and IH-L: conceptualization and original draft preparation. SB-B, AmB, GB, J-PG, MY, AlB, SB, and IH-L: writing–review and editing. All authors read and agreed to the published version of the manuscript.

## Conflict of Interest

The authors declare that the research was conducted in the absence of any commercial or financial relationships that could be construed as a potential conflict of interest.
